# Liquid Biopsy in Prostate Cancer Management—Current Challenges and Future Perspectives

**DOI:** 10.3390/cancers14133272

**Published:** 2022-07-04

**Authors:** Felice Crocetto, Gianluca Russo, Erika Di Zazzo, Pasquale Pisapia, Benito Fabio Mirto, Alessandro Palmieri, Francesco Pepe, Claudio Bellevicine, Alessandro Russo, Evelina La Civita, Daniela Terracciano, Umberto Malapelle, Giancarlo Troncone, Biagio Barone

**Affiliations:** 1 Department of Neurosciences, Reproductive Sciences and Odontostomatology, University of Naples “Federico II”, 80131 Naples, Italy; felice.crocetto@unina.it (F.C.); fmirto22@gmail.com (B.F.M.); alessandro.palmieri@unina.it (A.P.); biagio.barone@unina.it (B.B.); 2 Department of Public Health, University of Naples Federico II, 80131 Naples, Italy; gianlucar93@libero.it (G.R.); pasquale.pisapia@unina.it (P.P.); francesco.pepe4@unina.it (F.P.); claudio.bellevicine@unina.it (C.B.); umberto.malapelle@unina.it (U.M.); giancarlo.troncone@unina.it (G.T.); 3 Department of Medicine and Health Sciences “V. Tiberio”, University of Molise, 86100 Campobasso, Italy; 4 Medical Oncology Unit, Papardo Hospital, 98121 Messina, Italy; alessandrorusso@aopapardo.it; 5 Department of Translational Medical Sciences, University of Naples “Federico II”, 80131 Naples, Italy; eva.lacivita@gmail.com (E.L.C.); daniela.terracciano@unina.it (D.T.)

**Keywords:** liquid biopsy, prostate cancer, cancer biomarkers, circulating tumor cells, extracellular vesicles, cell-free nucleic acids, circulating nucleic acids, cell-free DNA, cell-free RNA

## Abstract

**Simple Summary:**

Prostate cancer (PCa) is a widespread malignancy, representing the second leading cause of cancer-related death in men. In the last years, liquid biopsy has emerged as an attractive and promising strategy complementary to invasive tissue biopsy to guide PCa diagnosis, follow-up and treatment response. Liquid biopsy is employed to assess several body fluids biomarkers, including circulating tumor cells (CTCs), extracellular vesicles (EVs), circulating tumor DNA (ctDNA) and RNA (ctRNA). This review dissects recent advancements and future perspectives of liquid biopsy, highlighting its strength and weaknesses in PCa management.

**Abstract:**

Although appreciable attempts in screening and diagnostic approaches have been achieved, prostate cancer (PCa) remains a widespread malignancy, representing the second leading cause of cancer-related death in men. Drugs currently used in PCa therapy initially show a potent anti-tumor effect, but frequently induce resistance and PCa progresses toward metastatic castration-resistant forms (mCRPC), virtually incurable. Liquid biopsy has emerged as an attractive and promising strategy complementary to invasive tissue biopsy to guide PCa diagnosis and treatment. Liquid biopsy shows the ability to represent the tumor microenvironment, allow comprehensive information and follow-up the progression of the tumor, enabling the development of different treatment strategies as well as permitting the monitoring of therapy response. Liquid biopsy, indeed, is endowed with a significant potential to modify PCa management. Several blood biomarkers could be analyzed for diagnostic, prognostic and predictive purposes, including circulating tumor cells (CTCs), extracellular vesicles (EVs), circulating tumor DNA (ctDNA) and RNA (ctRNA). In addition, several other body fluids may be adopted (i.e., urine, sperm, etc.) beyond blood. This review dissects recent advancements and future perspectives of liquid biopsies, highlighting their strength and weaknesses in PCa management.

## 1. Introduction

Prostate cancer (PCa) affects millions of men worldwide, representing the second most common type of malignancy in men, with 1.4 million of newly diagnosed cancers per year, and one of the leading causes of cancer-related death in men, accounting for 350,000 deaths per year globally [1,2].

In developed and industrialized countries, the incidence of PCa increases progressively with the age of the worldwide population. It has been estimated, indeed, that all-age incidence was 31 per 100,000 males, with a lifetime cumulative risk of 3.9% and more than 1 in 4 men over 75 years is affected by PCa [3,4]. PCa shows an extreme geographical variation both in incidence and mortality rates, being widely spread in developed countries (such as Europe, the United States of America, Canada, Australia and Middle-Southern Africa), while it is less common in developing ones. These differences could be mostly related to disparities in diagnostic tests frequency and potency among countries as well as lifestyle factors, as evidenced by migration studies [5]. An emblematic study by Shimizu et al. showed how an increased PCa incidence and mortality rate was observed among men migrating from Asian countries with a low-risk of PCa onset to European and North American countries with a high PCa risk, compared to men remaining in their native countries [6,7].

Nevertheless, despite the widespread prevalence of this disease, about 80% of cancers at diagnosis are limited to the anatomical bounds of the prostate gland with an estimated life expectancy of localized PCa patients up to 99% over 10 years [4,8]. However, on the other side, a minority of patients have local positive lymph nodes (about 15%) or distant metastasis (5%) at the diagnosis, reducing the 5 years survival rate at 30–40% [9].

Although PCa etiology is still not yet fully understood, it is recognized that both environmental (modifiable) and innate factors (unmodifiable) play a pivotal role in PCa onset [10].

Among unmodifiable factors, age is strongly and linearly associated with the PCa risk [11]. Similarly, Afro-Americans show an increased PCa risk, due to high levels of serum testosterone and insulin-like growth factor-1 (IGF-1) [12].

Finally, about 9% of PCa are hereditary forms, i.e., the affected patients have at least two relatives with a PCa diagnosis before the age of 55. Interestingly, genes involved in DNA damage repair mechanisms, are involved in PCa, such as *BRCA 1/2*, *HOXB13* and *RNaseL* (1q24-25) [13,14,15].

Among modifiable factors, the dysregulation of hormonal pathways, due to several environmental factors, such as metabolic syndrome, obesity, hypercholesterolemia and processed foods intake, leads to increased serum insulin levels, inflammatory cytokines and estradiol, which predisposes to an increased high-grade PCa risk [16,17,18,19,20,21,22].

The current clinical approaches in PCa diagnosis include digital rectal examination (DRE), prostate-specific antigen (PSA) measurement, imaging (transrectal ultrasound and multiparametric magnetic resonance imaging of the prostate) and prostate biopsies [23].

Although inexpensive, easy to perform and relatively noninvasive, the effectiveness of DRE, with a predictive positive value between 5% and 30%, is contingent on the experience and skill of the examiner [24]. Conversely, PCa diagnosis has been revolutionized by the introduction of serum PSA testing, being an early, comfortably and relatively inexpensive marker. However, PSA is an organbut not a cancer-specific marker, whose expression level is influenced by age and increases also in non-malignant conditions (e.g., benign prostatic hyperplasia, prostatitis, genito-urinary infections, DRE). Furthermore, the PSA cut-off level is still not standardized, and despite its role as PCa independent predictor, its use alone could be misleading [25,26,27,28]. PSA sensitivity ranges between 67.5% and 80%, while specificity is up to 40%. Therefore, about 20–30% of PCa could not be diagnosed if PSA is used as the only diagnostic test. To address this need, several new laboratory tests have been developed, with a clear tendency to combine panels biomarkers. Among these, the most promising laboratory tests are Phi (Beckman Coulter s.r.l., Milano, Italia) 4K score (BioReference Laboratories, Inc. Elmwood Park, NJ, USA) and Stockholm 3 (A3P Biomedical AB, Stockholm, Sweden) as circulating biomarkers, Mi-prostate score (MLabs, Ann Arbor, MI, USA), Exo DX Prostate (Exosome Diagnostics, Martinsried, Germany) and Select MD-X MDxHealth, Irvine, CA, USA as urinary biomarkers and Confirm MDx (Veracyte Headquarters, South San Francisco, CA, USA) Oncotype Dx (Exact Sciences, London, UK,), Prolaris (Myriad Genetics Corporate Headquarters, Salt Lake City, UT, USA) and Decipher (GenomeDx Biosciences, San Diego, CA, USA) as tissue biomarkers. These tests aimed to minimize overdiagnosis without missing the identification of clinically significant PCa [29].

Regarding the imaging, the use of the standard transrectal ultrasound sonography (TRUS) alone, albeit having improved the diagnostic capabilities in urological clinical practice, prior to the introduction of multiparametric magnetic resonance imaging (mpMRI), is still not reliable in detecting PCa, due to its limitations in recognizing only hypoechoic lesions in the peripheral zone of the prostate [30].

The mpMRI scan represents the game-changer of PCa diagnosis, due to its high sensitivity and specificity, reporting a negative predictive value between 92% and 100% for clinically significant tumors. In addition, mpMRI provides detailed anatomical and functional information on the prostate via the use of several standards weighed sequences, such as T1 (T1w), T2 (T2w) and diffusion (DWI), permitting to evaluate also the potential capsular and seminal vesicles infiltration of PCa. Nevertheless, the main limitations of the mpMRI are the high cost of this equipment and the limited number of radiologists experts in its interpretation [31,32].

Prostate biopsy represents the only procedure which allows a certain diagnosis and it is currently performed, under ultrasound guidance, transperineally or transrectally. A combined approach involving the use of coupled TRUS and mpMRI imaging (Fusion biopsy), has permitted to increase the overall accuracy of PCa diagnosis, especially in biopsy-naïve patients, reaching concordance rates with the definitive histologic report up to 52.3% (for targeted biopsy) and 85.5% (for systematic biopsy) [33].

Nevertheless, this approach shows several risks, such as hematuria, hematochezia and hematospermia up to a month after examination, increased body temperature, abscesses, bacteriemia, sepsis or lesions of the prostatic urethra and urinary retention [34,35].

Consequently, less-invasive methods aimed to reduce biopsy complications without lowering the detection rate of the procedure, are strongly needed.

**Figure 1:**
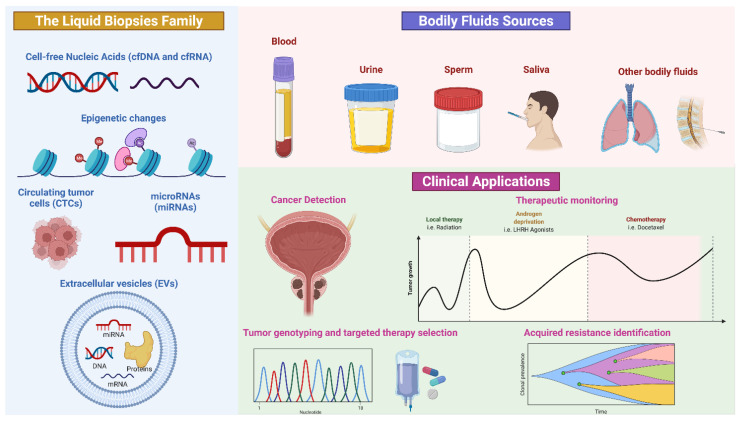
Schematic representation of liquid biopsy composition and application. Credit: Created with BioRender.com (accessed on 3 June 2022).

In the past few years, liquid biopsy has emerged as a new diagnostic and prognostic tool to trace cancer [36,37]. The term “liquid biopsy” refers, indeed, to a non-invasive analysis of biomarkers in biological fluids (such as blood, plasma, urine, liquor and saliva) to allow the detection, and the longitudinal follow-up, of cancers, avoiding the limitations of invasive procedures and, contextually, obtaining enough molecular information than those derived from tissue biopsies (Figure 1) [38].

The biomarkers commonly obtained from a liquid biopsy are circulating cell-free tumor DNA (ctDNA), circulating cell-free tumor RNA (ctRNA), proteins, peptides, metabolites, circulating tumor cells (CTCs) and extracellular vesicles (EVs), which incorporate genomic, epigenomic, transcriptomic and proteomic information of tumors. Furthermore, a single specimen could be used in multiple assays [39,40].

**Figure 2:**
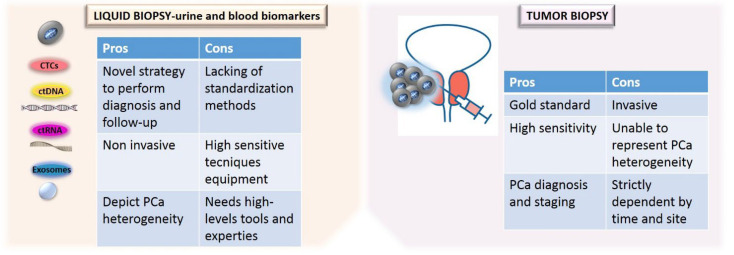
Comparison of the advantages and limitations of tissue versus liquid biopsy.

Another advantage of circulating biomarkers’ analysis is related to the reduction of intra-tumor heterogeneity, permitting to overcome the variability of molecular information obtained by tissue analysis which could be dependent on tumor localization and accessibility. Moreover, liquid biopsy displays the tumor microenvironment behavior. Finally, liquid biopsy provides a tool for monitoring tumor progression, predicting prognosis, overall survival and treatment efficacy, dictating a tailored therapy [41]. Figure 2 shows the advantages and limitations of tissue versus liquid biopsy (Figure 2).

This current review aims to summarize the potential implications of circulating serum and urine biomarkers analysis in PCa management, delineating current challenges and perspectives of the employment of liquid biopsy in clinical practice.

## 2. Blood and Serum Biomarkers in the Detection of PCa

The limitation met in the recovery of tissue biopsy highlighted the necessity to implement alternative biological sources [42,43]. The introduction in routine diagnostic practice of highly sensitive techniques encouraged the comprehension of tumor landscape, analyzing circulating tumor nucleic acids (ctNA), circulating tumor cells (CTCs) and tumor-derived extracellular vesicles (EVs) released by cancer cells by using blood samples [43,44,45,46]. A comprehensive table summarizes the blood, serum and urinary biomarkers reported in this review (Table 1).

### 2.1. ctDNA

Circulating cell-free DNA (cfDNA) analysis has gained relevance also in the setting of PCa. cfDNA represents DNA fragments released in blood by normal and tumor cells [66]. Remarkably, DNA released by tumor cells represents a small fraction of cfDNA, called ctDNA, which shows a smaller size than cfDNA released by normal cells [67,68]. From a prognostic point of view, ctDNA concentration in blood could potentially be complementary to PSA tests or replace it. High ctDNA concentration, indeed, correlates with poor PCa outcome [69]. Corbetta et al. reported a transient ctDNA concentration and fragment lengths increase after prostate biopsy at different time points [48]. Recently, Chen et al. have demonstrated that advanced stage PCa patients have a higher ctDNA concentration compared to those with localized disease or healthy controls. In this study, ctDNA was quantified with a Qubit 3.0 fluorometer and a DNA dsDNA HS Assay Kit (Life Technologies, Carlsbad, CA, USA), and the 2100 Bioanalyzer with High Sensitivity DNA Chips (Agilent Technologies, Santa Clara, CA, USA) was applied to assess purity, concentration and fragment size of sample analyzed [47]. In addition, the authors highlighted that ctDNA amount was remarkably increased (from 3.9- to 164-fold) after the surgical approach. Moreover, it was also estimated that cfDNA was characterized by a larger fraction of di-, tri- and multi-nucleosome associated DNA fragments [47]. Similarly, Kwee et al. observed, by RT-PCR analysis of the methylated promoter of the PCa-related genes *GSTP1* and *RARB2,* a significant ctDNA concentration increase after chemotherapy [49]. In fact, it has been demonstrated that specific hypermethylation of *RARB2* and *GSTP1* CpG sites may be adopted for PCa diagnosis [70]. According to cfDNA level modification as a clinical biomarker in PCa patients, in another experience, Patsch et al. evaluated a rapid decline of ctDNA amount quantified for long interspersed nuclear elements (LINE1) with qPCR approach after chemotherapy [50]. The phase III FIRSTANA and PROSELICA clinical trials revealed that ctDNA concentration may be considered an independent prognostic biomarker in advanced stage PCa. A higher ctDNA baseline concentration has been, indeed, associated with shorter progression-free survival (PFS) and overall survival (OS) after chemotherapy. Conversely, a total ctDNA concentration reduction during the first 9 weeks of treatment correlated with drug response therapy [51]. ctDNA analysis could represent a valid cost-effective alternative to tissue biomarkers analysis in advanced stage PCa. Interestingly, this approach could be useful to identify predictive biomarkers that can be further assessed in future clinical trials [67]. As an example, Wyatt et al., by comparing PCa ctDNA alterations with matched tissue, detected several genetic alterations, including Androgen Receptor (*AR*) amplifications, *SPOP* mutations and *TP53*, *PTEN*, *RB1*, *APC*, *CDKN1B*, *BRCA2* and *PIK3R1* genes inactivation, which may be further studied in these patients from a predictive point of view. In this setting, the remarkable concordance of ctDNA and metastatic tissue biopsies in advanced stage PCa patients suggests that ctDNA assays could be used for molecular stratification of patients for prognostic and predictive purposes [52,71].

### 2.2. ctRNA

Similarly to DNA fragments, tumor cells shade RNA-derived fragments in blood, known as circulating tumor RNA (ctRNA), ctRNA- messenger RNA (mRNA), microRNA (miRNA) and long non-coding RNA, may similarly represent a fascinating biosource for molecular analysis. In particular, the miRNAs expression profiling analysis is increasing to perform diagnosis, staging, progression, prognosis and treatment response [72,73]. miRNA can be extracted from ribonucleoprotein complexes or EVs [72,74]. Mitchell et al. firstly demonstrated the presence of miRNA in the plasma of PCa patients [75]. Since then, a large number of miRNAs were shown to be deregulated in PCa patients; in particular, miR-21, miR-30c, miR-125b, miR-141, miR-143, miR-148a, miR-205, miR-221 and miR-375 [76]. Liu et al., in 2018, performed a RT-PCR analysis of plasma samples collected from a cohort of *n* = 229 PCa patients on active surveillance, identifying three miRNA (miR-24, miR-223, and miR-375) that were significantly expressed in tumor patients. The authors elaborated two multi-variable logistic regression models, integrating the 3-miR score, PSA, the percentage of tumor cells in diagnostic samples and clinical variables. They showed that the 3-miR score ability to predict reclassification was not related to clinical variables and increased in comparison with clinical outcomes.

The authors concluded that the 3-miR score combined with PSA may represent a non-invasive high negative predictive value tool to identify patients on active surveillance who have indolent PCa [53]. Alhasan et al. identified in circulating miRNAs (miR-200c, miR-605, miR-135a, miR-433, and miR-106a) a molecular signature to detect high-risk PCa [54]. In 2017, Ferreira de Souza et al. analyzing plasma mRNA and miRNA of 102 untreated patients with PCa and 50 healthy subjects, identified differentially expressed *OR51E2* (olfactory receptor, family 51, subfamily E, member 2) and *SIM2* (single-minded 2) mRNAa, miR-200b and miR-200c. In addition, they showed that the *OR51E2* and *SIM2* genes association with miR-200b and miR-200c could be a diagnostic marker able to discriminate PCa samples from healthy controls with a sensitivity of 67% and specificity of 75% [55].

### 2.3. CTC

Circulating tumor cells (CTCs) originating from primary tumor are detectable in blood or lymphatic fluid [77]. Nevertheless, the use of CTCs for diagnosis is limited by the rarity of this cell population in blood [78]. In 2020, Ried et al. tested 20 CTCs samples from PCa patients, obtained with ISET^®^-CTC methodology, using the Immuno-Cyto-Chemistry staining (ICC) with PSA and protein antibodies, showing a positive result in almost all of the patients (18/20). In addition, in 27 early-stage patients, CTCs were found in 25 cases and 20 out of them had ICC-PSA-positive markers. Thus, a 99% positive predictive value and a 97% negative predictive value have been highlighted for the ISET-CTC-ICC approach [56]. Over the years, the importance of CTCs detection has also acquired clinical relevance as a prognostic and predictive biomarker [79]. Prospective trials showed that patients with an increase in CTCs amount within four weeks after chemotherapy could not benefit from treatment [57]. In 2021, Scher et al. displayed that the identification of CTCs, through the Epic Sciences platform, represents a prognostic biomarker for the progression of metastatic castration-resistant PCa (mCRPC) starting a second-generation androgen receptor signaling inhibitor (ARSI) [80].

### 2.4. EVs

In cancer development, EVs play a pivotal role in the signaling pathway network between tumor cells and the microenvironment [81,82]. In metastatic PCa patients, EVs promote metastasis by establishing the pre-metastatic niche (PMN). In fact, exosomes containing miRNAs (miR-21 and miR-139) promote PMS modifications [83]. For these reasons, EVs can have diagnostic and prognostic value in PCa patients. Several studies demonstrated that exosomes are more numerous in PCa patients than in healthy individuals [58,59,60,84]. However, according to Gao et al., nowadays, there are no standard methods to collect and analyze samples, rendering clinical and preclinical data inconsistent [81].

## 3. Urine Biomarkers in the Detection of PCa

Urine may be considered a suitable integrating source of clinical biomarkers that could play a pivotal role in the diagnosis, prognosis and PCa patients management [85]. From urine samples, various analytes may be isolated and detected. Among them, ucfDNA/RNA, miRNA, circulating tumor cells (CTCs) and extracellular vesicles (EVs) play a promising role in the clinical management of urogenital malignancy patients [86]. Urine cell-free DNA (ucfDNA) has recently been investigated in order to identify a novel potential biological source of nucleic acids able to integrate circulating nucleic acids from plasma samples in urogenital malignancy patients [87].

Remarkably, molecular analysis of urine analytes is characterized by several advantages: non-invasive sampling, with high volume of reproducible samples available in all time points with respect to low compliant sampling preparation [88]. Urinary biomarkers useful to predict biopsy outcome are often unimodal; a single urine fraction (i.e., cell-free fractions or cell-pellet) or biological cancer characteristic are considered to evaluate PCa status. Although a single test shows the accuracy and promising clinical relevance, the integration of multiple types of information could display a higher predictive value. ExoGrail is a multivariable risk model that integrate information from different clinical parameters. ExoGrail combines the expression level evaluation of Engrailed-2 (EN2), a protein contained in vesicles actively secreted by PCa cells and detected in urine samples with data from urinary cell-free RNA measurement. ExoGrail could be useful to assess PCa risk-assessment prior to an invasive tissue biopsy [89].

### 3.1. ctDNA

Based on recent literature data on the ctDNA fragmentation index in solid tumor patients, Casadio et al. carried out a pilot study on a retrospective series of bladder and prostate tumor patients aimed to technically validate the implementation of ucfDNA fragmentation index as a screening tool in PCa cohort [90]. Overall, it has been shown that urine DNA integrity is capable of distinguishing between PCa patients and healthy individuals with an accuracy of about 80% [61]. Moreover, Salvi et al. compared ucfDNA fragmentation index between *n* = 67 prostate malignant lesions and *n* = 64 benign prostate lesions grading in illness severity. Molecular data were obtained from a qPCR analysis of three oncogenic sequences longer than 250 bp (*c-MYC*, *HER2* and *AR*). Results showed a lower clinical predictive value than PSA in terms of sensitivity (0.58 vs. 0.95) and specificity (0.44 vs. 0.69), respectively [62]. In this context, PCA3 represents the first urine long noncoding RNA biomarker identified and approved by Food and Drug Administration (FDA) that could improve the detection rate of PCa [91]. Despite an increasing specificity, the quite low sensitive rate highlighted the necessity to discover other targets [92]. The expression of aberrant RNA transcript (*TMPRSS2: ERG*) represents a pathogenic mechanism in the development and progression of PCa [93]. Several studies have elucidated the prognostic role of residual or persistent *TMPRSS2-ERG* gene fusion expression in patients with castration resistant PCa [93,94]. A qRT-PCR analysis performed to detect *TMPRSS2: ERG* gene rearrangement in a retrospective series of *n* = 19 PCa patients (*n* = 11 prebiopsy and *n* = 8 pre-radical prostatectomy samples, respectively) revealed that 8 out of 19 (42.0%) PCa patients showed a detectable *TMPRSS2: ERG* aberrant gene fusion expression. In addition, it has been calculated the qRT-PCR sensitivity for urine *TMPRSS2: ERG* rearrangement detection by performing a Fluorescent in situ Hybridization (FISH) assay on corresponding PCa specimens. In this setting, FISH detected *TMPRSS2: ERG* in three patients with high frequency detected mutation from urine samples, while also highlighting a positive result in two patients negative for *TMPRSS2: ERG* gene fusion detection in ucfRNA specimens [63]. Accordingly, the implementation of the urine-based biomarkers in clinical practice was optimized with the diffusion of commercially available tests (IntelliScore -Exosome Diagnostics, Waltham, MA, USA and SelectMDx- MDxHealth, Irvine, CA, USA) aimed to determinate PCa patients selected for required tissue biopsy. In the era of “multi-omics” analysis, the development and diffusion of ultra-deep highly sensitive platforms, allowing to measure low target concentration in scant starting samples, have revolutionized the testing strategies in the clinical practice of tumor patients [95,96,97]. In an ongoing clinical trial promoted by the American Society of Clinical Oncology Genitourinary (ASCO-GU) an NGS assay, able to cover hot spot mutations in *n* = 152 cancer-related genes (PredicineCARE™, Predicine, Hayward, CA, USA), was used on blood and urine-derived circulating nucleic acids from *n* = 59 treatment-naïve PCa patients. Molecular profiling was then compared with corresponding data obtained from gold standard tissue specimens. Preliminary data elucidated a similar mutation profile between urine and corresponding tissue specimens with a sensitivity of 86.7% [98].

### 3.2. ctRNA

Recently, novel small non-coding RNAs have been investigated as promising diagnostic biomarkers for PCa patients [99,100]. Small RNA harbored by extracellular vesicles (EVs) could be considered a valuable marker for PCa diagnosis. Mckiernan et al. collected urine specimens from *n* = 1563 subjects. After a validation study aimed to evaluate gene expression signature in three genes (*PCA3*, *ERG* and *SPDEF*) involved in PCa progression, they focused on *n* = 255 not biopsied PCa patients with PSA level >2. The exosomes-derived gene expression profile showed a higher predictive value than PSA (AUC 0.73; 95% CI, 0.68–0.77 vs. AUC 0.63; 95% CI, 0.58–0.68) in the identification of high-grade PCa patients with respect to intermediate positive and negative biopsy from PCa patients. In addition, gene expression signature from urine exosomes also demonstrated a reliable clinically relevant predictive role (NPV 91.0%) in the decision making of patients with negative histological results [64]. Interestingly, the EPI urine biomarker was significantly associated with low-risk disease, making it a good test to select patients for AS [101].

### 3.3. CTC

Another approach to improve the diagnostic stage in PCa patients is based on the evaluation of circulating tumor cells (CTCs). The unique technical strategy approved by FDA for the detection of CTCs in peripheral blood of advanced solid tumor patients is the CellSearch test, able to detect (≥2 CTCs in 57% of metastatic PCa patients). In addition, CTC isolation from biological fluids have been recently improved with the implementation of microfluidic technology [102,103]. This technology provides a high-throughput and low-cost analysis and allows accurate CTC separation by cell size in an inert matrix [65,104]. CTCs isolation, confirmed by fluorescent staining (GPC-1^+^), was observed in 12 out of 14 patients (86.0%) while CTCs detection was negative in 11 out of 14 control group patients (79.0%). In the remaining cases, a weak GPC-1^+^ positive signal showed <8 CTC correctly detected. In addition, a positive correlation between GPC-1^+^ positive CTCs and PSA level was observed (r = 0.27) [105].

## 4. The Role of Liquid Biopsy in Follow-Up

PCa is commonly considered a “hormones-dependent disease”, since androgen controls PCa initiation and progression. Androgen deprivation therapy (ADT) represents the first-line therapeutic choice. Although ADT is effective to block tumor growth, this strategy often fails. Monitoring treatment efficacy represents a relevant aspect; currently, serum PSA and imaging are applied to follow treatment efficacy in PCa. However, the evaluation of early bone metastasis using imaging methods remains challenging, and PSA levels may be affected by AR signaling inhibitors. PCa often gains androgen independence, known as castration-resistant PCa (CRPC), characterized by metastatic spreading, significant mutational burden and copy number alteration, poor prognosis and a low survival rate [106]. CRPC often spreads in multiple sites per patient. Nowadays, despite several treatment options being available with varied mechanisms of action suitable for CRPC, long-term complete regression of CRPC is a rare phenomenon [107]. CRPC could depend to the transcriptional activity reactivation of androgen receptor (AR), because of *AR* gene mutations or amplification, leading to antiandrogens or other steroids promiscuous binding, or *AR* splice variants constitutively activated [108,109]. Since some tumors exhibit acquired resistance to specific chemotherapy agents could be possible to maximize the therapeutic efficacy by characterizing the tumor signature throughout the treatment. In this scenario, liquid biopsy has an advantage over tumor biopsy to capture genomic events from distant clones that are driving tumor progression [110]. Liquid biopsy may be used to early detect and manage a chemoresistance before the treatment pressure selects the most aggressive subclone of the tumor making it prevalent in tumor tissue. It has been demonstrated that the exosome-RNA and CTC isolated by plasma samples could be used to detect the androgen receptor splicing variant 7 (AR-V7), a predictive variant of resistance to AR signaling inhibitors. Furthermore, Tagawa and coworkers showed that the absence of the same variants in mCRPC CTC patients may be associated with better taxane treatment outcomes [111,112,113]. In addition, liquid biopsy could be also used to predict resistance to PARP inhibitors (PARPi), which are approved for treatment or maintenance therapy for several malignancies, including PCa. Tumors with somatic or germline *BRCA* mutations may be responsive to PARPi and platinum chemotherapy; liquid biopsy in this case can detect an acquired *BRCA* reversion associated with a poor response to PARPi [114]. In conclusion, given the high mutational burden characterizing CRPC, liquid biopsy may be a useful tool for early detection of tumor driving mutation, which eventually leads to chemoresistance and tumor progression. In this scenario, the follow-up using longitudinal analysis with liquid biopsy approach allows both the quantitative tracking of tumor burden to monitor treatment response and the assessment of clonal evolution by comparing genomic profiles over time.

## 5. Perspectives, Limitations and Future Perspectives

Biomarkers development for precision, tailored medicine in PCa management could be accelerated by liquid biopsy. Moreover, liquid biopsy could implement genomic testing into routine clinical practice, providing signatures of metastatic sites. The CTC counts, circulating nucleic acids amount and fragmentation, the ctDNA methylation status, represent prognostic and response biomarkers that could potentially guide therapeutic decisions in clinical practice. However, it should be noticed that liquid biopsy assays require analytical validation and should be clinically qualified for endorsement in routine clinical use. In this context, further evaluation in clinical trials and wide prospective studies are required. In addition, high cost, technology access and wide heterogeneity in definitions and isolation platforms impact the introduction of these biomarkers in routine clinical testing. The EVs use in a clinical setting is promising, but the standardization of isolation and application methods is challenging. Although liquid biopsy shows the significant potential to track the PCa clonal evolution that could be helpful to design an adapt, tailored therapeutic strategy to overcome cancer recurrence and increase the patient lifespan, developing liquid biopsy biomarkers still faces considerable challenges that hinder their clinical application. Firstly, despite the accessibility of powerful and high throughput tests, there is not enough evidence to support the routine use of liquid biopsy for early-stage cancer, making treatment decisions, monitoring, predicting response or for cancer screening. Secondly, the wide use of liquid biopsy in the clinical practice is still hampered by the costs and the limited knowledge of this technology in secondary centers. Indeed, liquid biopsy is too expensive for small centers to be used as a routine laboratory technique, with costs associated with equipment, reagents and properly trained personnel. Furthermore, in order to obtain the best results from liquid biopsy, a synergic work between urologists, oncologists and biochemist/bioinformatics is required during all the processes of this technology. Lastly, the post-processing laboratory work and statistical analysis needed are much more complex and time-consuming than the conventional pathology. As a result, also in this case, all the processes related to the comparison, interpretation and delivery of results have higher associated costs and resources consumption [115,116]. Despite the promising future of ctDNA as a driver of cancer treatment, several challenges need to be faced. There is a strong need to decrease costs and analysis time and to ameliorate the diagnostic performance for early cancer and minimal residual disease (MRD) detection. The technical challenges of turnaround time and costs will probably be addressed soon. The main barrier remains the clinical validation of ctDNA for the use as MRD and cancer screening biomarker. Currently, the liquid biopsy role in PCa management does not exceed the simple prognostic assessment. Thus far, the main issue to incorporate this approach in clinical decision-making is the lack of interventional studies demonstrating a clear advantage for the metastatic PCa patients. Further larger and long-term studies are required to assess whether ctDNA evaluation can be used for treatment-decision making. The identification of targetable alterations and emerging resistance biomarkers represents an attractive feature of liquid biopsy, particularly in CRPC, and could implement the precision medicine therapeutics in PCa. In the next years, the improvements of our knowledge in liquid biopsy application in decision-making strategy for mCRPC patients promise to revolutionize the mCRPC and dramatically improve the survival rate and quality of life of these patients.

## 6. Conclusions

PCa represent a major public health burden, whose incidence progressively grows. Although several progresses have been placed into investigating novel diagnostic and prognostic biomarkers for PCa, considering the inability of current biomarkers to predict disease aggressiveness, new efforts are needed to paint the intriguing PCa picture. Therefore, the discovery of novel and effective tools for early diagnosis, follow-up and prognosis in PCa patients is claimed. In this scenario, the liquid biopsy field in PCa has advanced exponentially, developing prognostic and predictive biomarkers and holding promise for a minimally invasive approach of monitoring tumor evolution. In this review, we described urinary and circulating biomarkers based on CTC, RNA and DNA as novel tools to improve the characterization and the treatment of PCa patients. These liquid biopsy biomarkers show the potential to gain comprehensive information on PCa genetic landscape, and give information about the metastatic sites. Liquid biopsy could guide therapeutic decisions and accelerate the development of precision medicine in PCa. The recent advancement of molecular biology techniques available will bring to the development of new standardized liquid biopsy tests with high sensitivity and specificity, and lower cost that could promote the diffusion of liquid biopsy in routine clinical practice. Designing a dynamic therapeutic strategy based on tumor features detected in real-time through the liquid biopsy could significantly improve the survival rate and the quality of life of PCa patients. Remarkably, nucleic acids extracted from biological fluids play a crucial role in the clinical management of PCa patients. Among conventional body fluids, peripheral blood still remains the most suitable source of nucleic acids, because a wide series of literature data critically evaluate the preclinical and analytical issues for blood-derived nucleic acids. Conversely, little was known about the use of nucleic acids purified from urine samples. However, due to their close connection with prostatic glands, further studies should be performed to evaluate the clinical meaning of biomarkers from urine samples.

## Figures and Tables

**Table 1:** Summary of blood, serum and urine biomarkers.

	Variables	Test Name	Manufacturer	Assay Type	Molecular Targets	References
**Blood Biomarkers**	ctDNA	Qubit 3.0 Fluorometer and dsDNA HS AssayKit	Life Technologies, Carlsbad, CA, USA	dsDNA Quantitation	dsDNA	[47]
	ctDNA	2100 Bioanalyzer with High Sensitivity DNA Chips	Agilent Technologies, Santa Clara, CA, USA	dsDNA Quantitation purity and fragment size	dsDNA	
	ctDNA	Fluorometer and Qubit™ dsDNA HS Assay Kit	Thermo Fisher Scientific, Waltham, MA, USA	dsDNA Quantitation	dsDNA	[48]
	ctDNA	Agilent High Sensitivity D5000 ScreenTape System on Agilent-4200 TapeStation	Agilent Technologies; Santa Clara, CA, USA	dsDNA Qualitative analysis	dsDNA	
	ctDNA	ABI 7900HT system	Applied Biosystems, Foster City, CA, USA	qPCR analysis of repeated genomic ALU sequences to detect and quantify cfDNA	dsDNA	[49]
	ctDNA	Microfluidic electrophoresis using the Agilent 2100 Bioanalyzer and High Sensitivity DNA Chips	Agilent technologies Inc., Palo Alto, CA, USA	DNA fragment length analysis	dsDNA	
	Gene promoters’ methylation	ND	ND	Sodium bisulfite-PCR	GSTP1, RARB2	
	ctDNA	iCycler iQ Real-Time PCR	Biorad, Hercules, CA, USA	qPCR analysis of long interspersed nuclear elements (LINE1) for ctDNA quantification	dsDNA	[50]
	ctDNA	Quant-IT Picogreen HS DNA kit and BioTek microplate spectrophotometer at 480ex/520em	Thermo Fisher, Waltham, MA, USA	dsDNA Quantification	dsDNA	[51]
	ctDNA	Illumina MiSeq (V3 600 cycle kit) or HiSeq 2500 (V4 250 cycle kit)	Illumina Inc., Towne Centre Drive, San Diego, CA, USA	ctDNA sequencing	AR, SPOP, TP53, PTEN, RB1, APC, CDKN1B, BRCA2, and PIK3R1	[52]
	ctRNA	ExiLENT SYBR^®^ Greenassay (Exiqon, Denmark) qPCR was performed on QuantStudio 6 Real-Time PCR System	Applied Biosystems, Foster City, CA, USA	qRT-PCR analysis	miR-141, 375, 21, 30c, 145, 26b, 223, 24, and let-7a	[53]
	ctRNA	TaqMan MicroRNA Assay, TaqMan PCR master mix and TaqMan probes. ABI Prism Model 7900 HT instrument was used to perform the qRT-PCR.	Applied Biosystems, Foster City, CA, USA	qRT-PCR analysis	miR-200c, miR-605, miR-135a, miR-433, and miR-106a	[54]
	ctRNA	Sso Advanced Universal SYBR Green Supermix (Bio-Rad, USA). The reaction was performed on the 7900HT Fast Real-Time PCR System Thermocycler	Applied Biosystems, Foster City, CA, USA	qRT-PCR analysis	OR51E2, SIM2	[55]
	CTC	ISET^®^-CTC Test and Immuno-Cyto-Chemistry (ICC)	Rarecells Diagnostics, Paris, France	immuno-cyto-chemistry	PSA	[56]
	CTC	CELLSEARCH assay	Menarini, Silicon Biosystems Inc., Bologna, Italy	immuno-cyto-chemistry	epithelial cell adhesion molecule (EpCAM), cytokeratins, CD45	[57]
	EV	CD63 Exo ELISA Kit (EXOEL-CD63A-1)	System Biosciences, Mountain View, CA, USA	ELISA	CD63	[58]
	EV	CD63 Exo ELISA KitEXOEL-CD63A-1); human glutamate carboxypeptidase 2 (FOLH1) ELISA kit (MBS901525)	System Biosciences, Mountain View, CA, USA;MY BioSource, Inc., San Diego, CA, USA	ELISA	prostate-specific membrane antigen (PSMA)	[58]
	EV	Mx-3000 or Mx 3005 instrument	Stratagene, Amsterdam, The Netherlands	qRT-PCR analysis for EV quantification		[59]
	CTC	CellSearch Instrument	Janssen Diagnostics Inc. Huntington Valley, PA, USA	CTC Enumeration	EpCAM+CK+CD45-	[60]
**Urine Biomarkers**	ctDNA	Qiamp DNA minikit; IQ SYBR green;Rotor Gene 6000 detection system	Qiagen, Milan, Italy; Biorad, Milan, Italy; Corbett Research, St. Neots, UK	qPCR analysis for ctDNA fragmentation index evaluation	c-Myc, BCAS1, HER2, STOX1	[61]
	ctDNA	Qiamp DNA minikit; IQ SYBR green;Rotor Gene 6000 detection system	Qiagen, Milan, Italy; Biorad, Milan, Italy; Corbett Research, St. Neots, UK	qPCR analysis for ctDNA fragmentation index evaluation	c-Myc, AR, HER2, STOX1	[62]
	ucfRNA	RNeasy Micro kit; Omni-Plex Whole Transcriptome Amplification (WTA) kit	Qiagen, Inc., Valencia, CA, USA; Rubicon Genomics, Ann Arbor, MI, USA	qRT-PCR	TMPRSS2:ERG gene fusion	[63]
	EV	ExoDx Prostate IntelliScore urine exosome assay; QIAGEN Rotor-Gene Q MDx System	Exosome Diagnostics, Waltham, MA, USA; Qiagen, Venlo, The Netherlands	qRT-PCR	ERG, PCA3, SPDEF	[64]
	CTC	MIL-38 immunofluorescence assay (IFA)	Minomic International Ltd., Sydney, Australia	immunofluorescence	glycoprotein glypican 1 (GPC-1)	[65]
